# Cultivating China’s Cinchona: The Local Developmental State, Global Botanic Networks and Cinchona Cultivation in Yunnan, 1930s–1940s

**DOI:** 10.1093/shm/hkz099

**Published:** 2019-10-23

**Authors:** Yubin Shen

**Affiliations:** Department III (Artefacts, Action and Knowledge), Max Planck Institute for the History of Science, Boltzmannstraße 22, Berlin, 14195, Germany

**Keywords:** cinchona cultivation, the developmental state, global networks, Yunnan

## Abstract

This article reconstructs the history of China’s first successful cinchona cultivation programme in Hekou, Yunnan province from the 1930s to 1940s during the Nationalist era (1928–49). I argue that the Hekou programme was initiated by the Yunnan ‘local developmental state’ to control endemic malaria and achieve quinine self-sufficiency. It was expanded during the Sino-Japanese War (1937–45) as part of the national defence project in order to develop Yunnan’s malaria-ridden southwest frontier to provide more resources for the war, as well as to solve broader wartime epidemic crises in southwest China. A closer examination also indicates that the development of the Hekou programme was closely intertwined with global networks of cinchona cultivation and international politics.

In its third issue published in late spring of 1937, the leading Chinese scientific journal *Kexue* excitedly reported that, Hekou County Experimental Farm for Tropical Plants in China’s southwest Yunnan Province, for the first time in Chinese history, had successfully cultivated several hundreds of cinchona trees.^1^ The breaking news was soon broadcast around China, as cinchona, containing the effective antimalarial alkaloid quinine in its bark,^2^ was one of the most valuable medicinal plants in the world. Moreover, it was very difficult to cultivate cinchona outside the plant’s native Andean regions of South America, and previously, only a few Euro-American countries and Japan had achieved cinchona cultivations. With the success of Hekou Farm led by Yunnan Provincial Commissioner of Reconstruction Zhang Banghan (1885–1958), China finally had its own cinchona trees, just like those Western powers.^3^

Based on the primary sources from the Yunnan Provincial Archives, this article explores the origin, development and demise of China’s first successful cinchona cultivation programme in Hekou, which lasted mainly from the 1930s to the 1940s. It argues that the Hekou case does not neatly fit the current dormant colonial paradigm of the history of cinchona, in which cinchona (and quinine) was regarded as a ‘tool of empire’,^4^ and cinchona cultivation as an ‘imperial science’^5^ or a ‘colonial botany’^6^ for Euro-American and Japanese imperial expansion and colonial rule in their malaria-ridden tropical colonies.

As its Western counterparts, the Hekou cinchona cultivation programme was not a pure scientific agricultural experiment, but it served for more complicated political and economic purposes rather than a simple colonial or imperial project: it was initiated in the early 1930s by Zhang Banghan and his colleagues, in order to control endemic malaria and save national revenue for building Yunnan governor and warlord Long Yun’s (1884–1962) ‘local developmental state’, which resembled the technocratic central government of Nationalist China (1928–49). After the outbreak of the Sino-Japanese War (1937–45), the Hekou programme was incorporated into the Chinese national defence project. In a certain form of ‘internal colonialism’, it was then advanced to develop Yunnan’s semi-autonomous and malaria-ridden southwest frontiers to provide more resources for wartime needs. For another, together with other state-led bioprospecting programmes, it was also promoted by the central government to solve broader wartime epidemic crisis in Southwest China. In addition, the war played a key role in destabilising the Hekou programme. A closer examination on its integrations into Yunnan’s local state building and Chinese national defence projects also indicates that the Hekou programme was intertwined with global networks of cinchona cultivation and international politics.

## The Local Developmental State, Malaria and Yunnan Initiatives

Cinchona barks were brought to China by Jesuit missionaries in 1693 in order to cure Kangxi (r. 1661–1722), the second emperor of Qing China who suffered a serious malarial fever. For the next hundred years, the knowledge and application of cinchona barks gradually spread beyond the imperial court.^7^ Since the mid-nineteenth century, isolated quinine pills had also been introduced and widely used in China along with European imperial expansions.^8^ Recognising the medicinal and economic importance of the plant, some Chinese initiatives had attempted to cultivate cinchona in several southern Chinese provinces during the 1920s, but all of them had failed. It was not until 1935 that cinchona trees were successfully cultivated in Hekou County Experimental Farm for Tropical Plants, which was initiated by Zhang Banghan and his colleague Huang Riguang of the Yunnan Commission of Reconstruction.

The Yunnan Commission of Reconstruction led by Zhang Banghan was a crucial part of the provincial administration of warlord Long Yun’s ‘local developmental state’. The term ‘developmental state’ was originally used by Chalmers Johnson to describe the Japanese state’s regulation and planning in Japan’s economic development.^9^ By applying this concept into modern Chinese history, scholars have already demonstrated that Nationalist China (1928–49) was an embryonic developmental state run by technocrats.^10^ Following its founder Sun Yat-sen’s (1866–1925) developmentalist plans,^11^ the Nationalist government appropriated the technocratic authority to legitimate its developmental policies in water control and national reconstruction.^12^ Some recent studies have already moved to look at local state building carried out by provincial authorities,^13^ such as warlord Chen Jitang (1890–1954) of Guangdong province in South China, who relied on agricultural experts to carry out his local developmental state project of the sugar industry.^14^ Long Yun ruled Yunnan in a manner consistent with his Guangdong counterpart. After he took control of this province in 1927, Long Yun called for ‘reconstructing a new Yunnan’ and established several new and effective economic, military, educational and public health institutions, including the Yunnan Commission of Reconstruction,^15^ and China’s first provincial governmental agency specifically designed to combat endemic malaria, the Yunnan Anti-Malaria Commission (YAMC) in 1939.^16^

Zhang Banghan was a lifelong disciple of Sun Yat-sen and a strong political supporter of Long Yun. Originally from Yunnan, Zhang joined Sun Yat-sen’s Anti-Qing Revolutionary Alliance in 1906. He was then sent by Sun to study in Europe in 1909 and later received a Belgian diploma in Electrical Engineering. After the establishment of the Nationalist regime in Nanjing in 1928, Zhang was appointed by Long Yun as the Provincial Commissioner of Reconstruction, a position he would hold until 1945.^17^ During his tenure, Zhang avidly sought to realise Sun and Long’s developmentalist ideas. In a special issue of the journal *Chinese Reconstruction* (*Zhonguo jianshe*) in 1934, Zhang elaborated his reconstruction plans for Yunnan, including promotion of modern farming and afforestation, as well as building modern transportation, hydraulic works and electricity systems. Considering the sub-tropical and tropical environmental conditions in south Yunnan, he proposed there should be farms for tropical cash crops, such as coffee, tung trees and plantains.^18^

His interest in agriculture and forestry was not exceptional. During the early twentieth century, many intellectuals and officials believed that in order to save China, it would be necessary to modernise Chinese agriculture and afforestation using the science of forestry.^19^ Since he did not know much about forestry himself, Zhang turned to Huang Riguang. Huang had graduated from the National Institute of Agriculture in Paris, and in 1924, he became a professor and chair of the experimental farm at the College of Agriculture of the newly established Guangdong University. The College of Agriculture, formerly an independent Guangdong Provincial Agricultural School, was incorporated into the university by Sun Yat-sen, who was convinced of the importance of modern agriculture, in order to build a strong focus on agriculture to ensure a better Chinese livelihood.^20^ It soon became one of the leading agricultural colleges in China, especially famous for forestry.^21^ In this particular college, Huang established himself as an expert on agriculture and tropical plants.^22^ Invited by Zhang, Huang went to Yunnan in 1928 to serve as director of the Forest Section in the Commission of Reconstruction.^23^

Both as technocrats trained in Europe, Zhang Banghan and Huang Riguang believed that by using modern scientific methods and rational management styles, they could more efficiently develop Yunnan. In the years that followed, they set up several institutes for the improvement of rice production, sericulture, tobacco, cotton, animal husbandry, tea, pest control and trees that could be grown for profit. They also established a provincial-wide system of tree farms for the purpose of afforesting Yunnan.^24^

It was Huang who first persuaded Zhang to cultivate cinchona trees in Yunnan.^25^ As early as 1924 back in Guangdong University, he had acquired dozens of cinchona seeds from overseas Chinese contacts in the Dutch Java, the dominant cinchona planter and supplier in the world. He started experimenting on the college farm but unfortunately failed in his attempts.^26^ Huang convinced Zhang of the importance of continuing his cinchona cultivation effort in Yunnan, using three major arguments: First, Yunnan, particularly its southwest frontier, had been a notoriously malaria-ridden region. At that time, over 10,000 patients died from malaria every year in Yunnan due to the shortage of quinine supplies. For example, Simao, a prosperous regional commercial centre in southwestern Yunnan, fell victim to a devastating malaria epidemic in 1919, lasting nearly 30 years. As a result, in 1927, its population had dropped from 76,800 to 24,106. After that, the population continued to decrease: in 1935, only 4,000 survivors lived there, and in 1953, Simao became little more than a ghost town, with only 1,092 residents.^27^ Thus, cultivating cinchona (to produce quinine) would save people’s lives,^28^ provide healthier labourers for Long Yun’s local developmental state and help strengthen his ruling legitimacy.

The second argument was saving national revenue. Malaria was endemic not only in Yunnan but also throughout China’s southern provinces. Zhang and Huang pointed out that 8–10 tons of quinine pills were imported from overseas every year, resulting in a huge loss of government funds. What is worse, the quantities of imported quinine were only sufficient to meet the needs of less than one-third of the total malarial patients in China, but increasing the import threefold would have meant yet more expense.^29^ According to another calculation in the 1930s, around 16 tons of quinine would have been required in order to treat all of the malarial sufferers in Yunnan. And throughout all of China, the total demand for cinchona bark, not to mention quinine products, was around 2 million *jin* (100 tons) every year, which would have amounted to a cost for the government of 150 million Chinese dollars (Table 1).^30^

**Table 1:** Quinine imports in China from the Dutch East Indies, 1935–39

Year	1935	1936	1937	1938	1939
Quantity (metric ton)	70	79	38	32	65

*Source*: From Nihon Yushutsu Nōsanbutsu Kabushiki Kaisha, *Beikoku o nayamasu fusoku shigen kina ni tsuite* (Tokyo, 1942), 34.

The idea of achieving autarky and building China’s national economy was widely accepted by officials in their national reconstruction projects.^31^ Zhang and Huang were among those technocrats who believed in these principles. They could not abide the fact that China was obliged to pay out huge sums for quinine imports, and thus, they planned to cultivate cinchona trees to save government expenditure, achieve quinine self-sufficiency, and possibly, in the process, gain considerable profits for the Chinese nation and the Yunnan local developmental state in the future.^32^

The final reason had to do with the fact that Yunnan possessed a potentially favourable climate and environment for growing cinchona trees. Cinchona with its strict environmental requirements is perhaps one of the most difficult plants to cultivate. According to contemporary Western scientific experts, cinchona trees with a high proportion of quinine would only grow at latitudes from 10° north to 20° south, at altitudes from 1,200 to 2,000 m, with average temperatures from 18°C to 22°C, annual rainfall from 2.5 to 3.5 m, with little monthly variation and humus-rich soil.^33^ Although Yunnan’s latitudes were from 20° to 28° north, taking into consideration its particular tropical climate and soil conditions, Zhang and Huang believed that cinchona trees could be cultivated in the province’s south and southwest malaria-ridden regions.^34^

## Global Botanical Networks and Cinchona Cultivation in Yunnan 

When Zhang Banghan and Huang Riguang started their cinchona programme in Yunnan in the 1930s, the first difficult task for them was to acquire cinchona seeds. Educated in Europe, they recognised that there were already extensive global botanical networks of cinchona cultivation in place they could turn to.

Global botanical cinchona cultivation networks were initiated by the British and Dutch empires in the mid-nineteenth century, joined later by other imperial powers who also participated in cinchona cultivation for their own specific colonial agendas.^35^ British colonial botanists and naturalists, such as Clements R. Markham and Richard Spruce, began to smuggle cinchona seeds out of South America and cultivated the plants in India in the 1850s, in order to safeguard British imperial enterprise in this malaria-ridden colony and to save huge expenditures on importing cinchona barks from South America. With support from the British Indian government and Kew Botanic Gardens, in a ‘global network of exploration collection and systematisation of botanical knowledge’,^36^ cinchona was successfully cultivated in places like the Nilgiri Hills in British India in the 1860s. After that, through Kew Garden’s ‘global reach and its connections with state power’ and its branch botanical gardens in various British colonies (including the Calcutta Botanic Garden in Bengal, which would play an important role in Yunnan’s cinchona cultivation), many cinchona plantations were established in India, East Africa, the Caribbean, Ceylon (Sri Lanka) and Burma.^37^

At almost the same time, in the 1850s, the Dutch government also started cinchona cultivation efforts in the Netherlands East Indies, particularly in Java, which boasted a similar topography and climate to South America. Under the direction of colonial scientists such as Franz Junghuhn and K. W. van Gorkom and with the Dutch government’s support, cinchona plantations in Java experienced rapid development. They began to enjoy huge economic profits, especially after 1872 when Charles Ledger’s seeds (*Cinchona ledgeriana*) were widely planted in Java.^38^ From 1890 onwards and up until 1940, the Netherlands East Indies gradually replaced British India and dominated the cinchona bark trade, by providing more than 90 per cent of the world’s supply.^39^

The French started their cinchona cultivation experiments in 1848 but soon faced failure. In 1869, seeds of cinchona trees were transported from Java to Saigon Botanic Garden in French Indochina. After several major failures in the five decades that followed, led by the famous bacteriologist Alexandre Yersin (1863–1943) of the Pasteur Institute in Saigon, cinchona trees were finally successfully cultivated in the 1920s in southern Vietnam.^40^ The German cinchona project was initiated in 1900 in the colony Tanganyika of East Africa, while the Americans cultivated cinchona trees in the Philippines in 1927.^41^ The Japanese Empire opened its own cinchona cultivation plan in the 1880s and succeeded in 1922 in its tropical colony Taiwan.^42^

The Yunnan initiatives understood that the global networks were of an imperial and colonial nature, but still, they struggled to take advantage of them. At first they tried to get cinchona seeds from Java, but for reasons still not clear, the Dutch colonial government seemed to have placed strict restrictions on the exportation of cinchona seeds to the Chinese in the early 1930s.^43^ After several failed attempts, in 1932, Zhang turned to Henry Forster Handley-Derry (1879–1966), the British consular official in Yunnan. Handley-Derry agreed to help Zhang and managed to purchase eight ounces of cinchona seeds from Java and Calcutta.^44^ Huang distributed some of these seeds to Lufeng Forest Farm,^45^ south of Yunnan’s capital Kunming, for a cultivation experiment, but they all died fairly soon. The rest of the seeds were sent to the Hekou Experimental Farm for Tropical Plants in Hekou County along the southwest borderland. Surprisingly, they grew very well, and the young plants soon began to leaf out. But the director of Hekou Farm, Zhang Jiliang, noticed that the leaves were completely different from the phyllotaxy of cinchona as described in textbooks. At that time, the famous American explorer and botanist Joseph Rock^46^ (1884–1962) was travelling in Yunnan. Huang Riguang asked him to examine the plants. The results were very striking: these plants turned out to be ficus (banyan), not cinchona at all! There were two possible explanations for this mix-up: either the suppliers had given ficus seeds in the first place, or the cinchona seeds had been stolen and replaced with ficus mid-journey somewhere in Vietnam. Whatever the explanation, this further supports indication that obtaining cinchona seeds was still not an easy task.

In the winter of 1933, Handley-Derry once again purchased eight ounces of cinchona seeds for Yunnan initiatives from the Calcutta Botanical Garden. This time, he carefully packed and delivered them in secure diplomatic bags, and Yunnan project finally obtained authentic cinchona seeds.^47^ Cinchona seeds from Calcutta were all brought to the Hekou Experimental Farm for Tropical Plants for cultivation. This farm was established in 1930 mainly for planting urban street trees,^48^ and in 1933, it was reorganised for experimenting with tropical plants such as coconuts, bananas and coffee.^49^ Zhang Banghan and his colleagues understood that the Hekou farm was not the perfect place to grow cinchona. The soil there was sandy loam, and its altitude was only 100 meters, which did not meet some of the critical requirements for cinchona cultivation. However, located on the border between Yunnan and Vietnam, it was the southernmost state-controlled farm and the only available one that was located around the latitude of 20° north in Yunnan. What is more, Hekou had certain climatic advantages that might favour the growth of cinchona trees, such as an average temperature above 21°C. For these reasons, the Yunnan initiatives still considered it to be worth a try.^50^

There was another possible advantage for cultivating cinchona trees at the Hekou Farm: the director and staff, trained as professional forestry experts, were strongly determined to transform Yunnan through means of modern agricultural science. The director, Zhang Jiliang, graduated from the School of Agriculture, Guangdong University, with a specialism in forestry.^51^ Lin Yongxin, the deputy director, was also an alumnus from that college. As Lin recalled years later, ‘to cultivate cinchona trees to save the patients’ lives’, they were ‘resolute in their determination’.^52^ Since ‘the foreigners kept the knowledge of cinchona cultivation as secrets’, they had little practical information or experience in cultivating this unfamiliar plant.^53^ They attempted to sow cinchona seeds eight times, but the trees all failed to sprout. Zhang Banghan encouraged them by citing the example of the drug Salvarsan, experiments with which had failed 606 times before this magic bullet against syphilis was created.^54^ On the ninth try, Zhang Jiliang and his staff in Hekou experimented with a technological innovation: they planted the seeds in boxes, and they finally germinated. Over 1,800 young plants were then successfully transplanted to the hills of the Hekou Farm. After 1936, they were even able to sow the seeds obtained from cinchona trees growing in the Hekou Farm itself.^55^ This piece of news was soon broadcast around China, and Zhang Banghan and Hekou Farm became well-known in China’s scientific and medical circles. It was widely believed that following the Hekou model, the Chinese would continue ‘acclimating cinchona trees to Chinese environments’.^56^

In the next years, Yunnan continued to acquire cinchona seeds through the global botanical networks. In 1939, the French government presented them with cinchona seeds as gifts.^57^ In 1940, the British consulate sent them seeds of red cinchona (*Cinchona succirubra*).^58^ The American consulate offered 5.6 g of three different types of cinchona seeds in 1942.^59^ By the 1940s, Yunnan had become part of the global network of cinchona cultivation.

## Cultivating Cinchona for the War: Developing the Pusi Frontier and Bioprospecting Anti-Malarial Drugs

The Yunnan state-building technocrats were not satisfied with their minor success in Hekou. They drafted ambitious plans to cultivate millions of cinchona trees in the province’s southwest region or the Pusi frontier, which seemed to boast more suitable land at higher altitudes. After the outbreak of the second Sino-Japanese war in 1937, two new motivations pushed them to put some of their plans into practice.

The first one was developing Yunnan’s Pusi frontier to supply resources and food for wartime needs. After the outbreak of the war, the Nationalist central government retreated to southwest China to ensure its survival. Over one million refugees from Japanese-occupied areas fled to Yunnan. To support the war and the huge population, it was critical to stimulate Yunnan’s economy further to provide more food and resources.^60^ As Long Yun claimed in a speech on the relationship between the war and developing Yunnan, the province boasted rich resources, such as water power and ‘uncultivated wasteland’, which could be better utilised to achieve final victory against the Japanese.^61^

Among these ‘fertile wastelands’, Pusi was regarded as ‘the perfect cultivable land for agriculture’,^62^ but it was a challenging mission to develop this ‘disobedient and dangerous’ frontier. The Pusi frontier was part of the so-called *Zomia* region, where non-Han indigenous regimes had maintained semi-autonomy away from the Chinese central government for hundreds of years.^63^ Ever since the late eighteenth century, the Qing government had substantially expanded its state power by transforming some of these local chieftainships into direct administrations and encouraging move ‘sophisticated’ Han Chinese peasants into this area to cultivate the land.^64^ However, the Pusi frontier was notorious for its serious malaria endemicity. As some scholars have pointed out, while the local ethnic Tai people were able to survive, having developed a hard-won resistance against malarial parasites, for most of the newly arrived Han Chinese immigrants, the disease had fatal consequences. This was the land where ‘no Han could go for long’.^65^ Such a long-term disease frontier environment had been maintained during the 1930s and 1940s, which posed a critical obstacle to the proposed cultivation plans of Long Yun’s local developmental state.^66^

The solution for eliminating malaria in Pusi frontier was to move more Han Chinese to reclaim land there during the Qing period. Republican intellectuals and officials held a similar view: ‘… there is a mutual waning and waxing relation between reclamation and malaria … if more people move in, and all wastelands are reclaimed, malaria endemicity will just go away’.^67^ It was similar as the ‘bonification’ measure promoted by European malariologists in the late 1920s, which ‘signified all work carried out with the object of making regions that are periodically or permanently marshy more healthy and more suitable for agriculture’, as the drainage and filling of swamps could eliminate breeding places of the malarial mosquitoes.^68^

After cinchona trees were successfully cultivated in Hekou, republican technocrats began to view cinchona tree plantations together with Han Chinese agricultural immigration as the best solution to develop the Pusi Frontier.^69^ Huang Riguang elaborated this point in a public lecture at Yunnan University in 1939: promoting the cultivation of cinchona was ‘the pioneering solution for reclaiming the malarial region’, since cinchona could protect Han Chinese immigrants against endemic malaria. For Huang, it became necessary to transplant cinchona trees in the counties of the Pusi frontier beyond Hekou.^70^ To collect more information about the Pusi frontier, and seek suitable places for planting cinchona trees there, Huang Riguang led the Investigating Team of the Pusi Frontier from November 1938 to February 1939, including Yuanjiang, Ning’er, Simao, Cheli, Fohai and Nanqiao Counties. During this trip, they left bottles of cinchona seeds and instructed local officials to try to cultivate cinchona trees there. The major success was achieved in Nanqiao. Zhang Jiliang brought cinchona seeds to Nanqiao Forest Farm, finding that its altitude of 1,400 meters was more suitable for cultivating cinchona, and thus a sub-programme was set up to cultivate cinchona there.^71^

The role of cinchona cultivation in developing the Pusi frontier to some extent resembled Euro-American and Japanese counterparts in their tropical colonies, but more precisely, it served as ‘a tool of internal colonialism’ rather than a ‘tool of empire’. As James Scott puts in his study on Zomia, ‘the encounter between expansionary states and self-governing peoples’ in this region was a ‘cultural and administrative process of internal colonialism that characterizes the formation of most modern Western, nation states’, which ‘involved a botanical colonization in which the landscape was transformed by deforestation, drainage, irrigation, and levees to accommodate crops, settlement patterns, and systems of administration’.^72^ The promotion of cinchona cultivation in the malaria-ridden Pusi frontier, together with Han Chinese immigrations during the wartime period, had undeniably shaped the ‘internal colonial’ nature of the Hekou programme.

Another important motivation was the central government’s need for anti-malarial drugs, following its retreat to malaria-infested southwest China. Even though the Allies and international organisations provided huge amounts of quinine pills, the total demand could not be met. There was a nation-wide, desperate search for Chinese anti-malarial drugs that might serve as a substitute for quinine, otherwise known as ‘bio-prospecting’,^73^ which also paralleled similar search projects undertaken elsewhere, such as in British India and post-colonial Africa.^74^ The most famous case was the promotion of the Chinese herb *changshan* (*Dichroa febrifuga*).^75^ After its efficacy against malaria was proved by Chinese scientists, the Nationalist central government set up several changshan cultivation projects in the 1940s.^76^

Nevertheless, within this context of seeking anti-malarial drugs, even more attention was paid to China’s own cinchona trees in Yunnan. As early as 1936, the Fourth Congress of the Chinese Medical Association had already proposed that the Nationalist central government promote cinchona cultivation in China’s tropical provinces—Guangdong, Guangxi, Yunnan and Guizhou—in order to treat malaria-infested patients, to save revenue and to prepare for a possible world war.^77^ In 1939, having heard the news about Hekou’s successful cinchona cultivation, the Congress of the Chinese Medical Association sent an official letter to inquire into the details of the Yunnan initiative’s success. The Yunnan Commission of Reconstruction saw it as a good opportunity to attract the central government’s attention and ordered the Hekou Farm to prepare reports of its cultivation achievements for both the Congress of the Chinese Medical Association and the central government.

Hekou Farm also tried to gain more funding by proving that their cinchona bark contained a high percentage of quinine alkaloids and was thus effective in practical treatment. In November 1939, they sent several bark samples to the Army Factory of Medical Materials, and the reports indicated that these samples contained 6.54 per cent quinine alkaloids, which reached the minimum level of 5.4 per cent required by the United States Pharmacopeia. In 1940, the farm sent another two samples to the Tonkin Institute of Chemical Analysis in Vietnam, and the results were also quite promising (Table 2). During malaria epidemics in Hekou in early 1940, they distributed some cinchona barks to local malaria patients and collaborated with the local malaria-control station of the YAMC to produce some quinine out of Hekou cinchona bark for malaria treatment. It was reported that many had been cured by taking Hekou-produced bark and quinine, and the staff proudly claimed that ‘cinchona bark from our farm is considerably useful’.^78^

**Table 2:** Dosage of alkaloids in Cinchona from Hekou farm (1940)

Cinchona species	Dosage of alkaloids in 3-year-old plants (percentage)	Dosage of alkaloids in 7-year-old plants (percentage)
*Cinchona ledgeriana*	3.00	13.97
*Cinchona succirubra*	2.56	9.52
*Cinchona malabar*	1.75	7.92

*Source*: From Yunnan Provincial Archives, 77-23-76, ‘Yunnan jianshe ting zhongzhi jinji’na zhi shikuang’.

**Table 3:** Cinchona trees cultivated in Hekou farm (1941)

Location	Planting time	Height (m)	Condition	Number
Mountain field	5/1934	4	Good	377
Mountain field	5/1939	1	Good	1,560
Nursery	5/1939	none	Good	32,000
Nursery	5/1940	0.1	Good	40,000
Baisa field	5/1934	6	Good	250

*Source*: From Yunnan Provincial Archives, 77-9-966-02, ‘Yunnan sheng jianshe ting Hekou redai zuowu shiyanchang 31 nian 1 yue gongzuo baogao’.

As a result, Hekou Farm soon enjoyed a boom in the number of visitors, especially those from the central governmental agencies, such as the Army Factory of Medical Materials, the National Drug Research Institute and the National Military Medical School. These visitors had been informed that the Hekou Farm could not promote massive cinchona cultivation due to the limited funds it received from the provincial government, and all agreed that the central government should provide more financial support. One of them even reported this situation back directly to Nationalist China’s highest leader at the time, Generalissimo Chiang Kai-shek (1887–1975), who then ordered the central government to allocate more funding in early 1940.^79^ It was also in 1940 that the National Congress proposed that the central government utilise special medicines produced in southwest China, in particular cinchona, to save more sick soldiers’ lives in order to strengthen China’s power against the Japanese.^80^

As a result of all these efforts, Yunnan’s cinchona cultivation programme was incorporated into the central government’s national defence project so that they began to receive sufficient funding. In light of this support, Yunnan state-builders thus drafted an ambitious plan, believing that there would be a promising future for their cinchona cultivation project: from 1941 to 1945, the Hekou Farm would cultivate millions of cinchona trees, set up eight new cinchona farms in southwest Yunnan within 5 years and even build a quinine factory in 1951, when there would be adequate grown cinchona trees available. In the first year of 1941, everything was going well: the mature trees in Hekou numbered 3,000, and there were more than 50,000 seedlings. Several new farms had already been established along the Pusi frontier, where cinchona trees had been successfully transplanted from Hekou (Table 3).^81^

However, this plan never came to fruition. After the Japanese successfully secured the occupation of French Indochina in late 1941, Hekou became a frontline zone directly facing the Japanese military threat. Its director and main staff retreated to Kunming in the Yunnan interior, and financial support from the central government was also cut off. As a result, the Hekou Farm ceased to exist except in name, and its cinchona cultivation programme was suspended in 1943 and was officially abandoned 2 years later.^82^ Only hundred or so of the surviving cinchona trees were then handed over to a local military garrison.^83^

The Hekou programme was once again restarted in 1948 by the new Yunnan governor Lu Han (1895–1974), when the Civil War (1946–49) between the Communists and the Nationalists had already broken out.^84^ Soon thereafter with the Nationalist’s defeat in 1949, the Hekou farm was taken over by the new Chinese communist regime. In 1953, the Hekou farm was transformed into a new experimental field for rubber plantations to supply ‘communist’ rubber to the Soviet Union, and its cinchona cultivation programme was entirely abandoned.^85^

## Conclusion

The past of China’s first successful cinchona cultivation programme in Hekou, Yunnan province, has been almost totally forgotten by historians of modern China in general, not to mention Chinese and global historians of medicine. By digging up primary archival sources in Yunnan Provincial Archives, this article reconstructs, for the first time in any language, the origins, developments and the demise of this cinchona cultivation programme between the 1930s and the 1940s, which adds to our understanding of the history of the global spread of cinchona in Nationalist China.

We have gained and are still gaining an increasingly comprehensive understanding of cinchona cultivation in Spanish, British and Dutch empires,^86^ but as Ya-wen Ku points out, the East Asian context has been largely overlooked.^87^ In her study on Japanese cinchona cultivation in Taiwan, Ku demonstrates the importance of the ignored private sector in the imperial-controlled enterprise. The present article, in elaborating the close relation between cinchona cultivation and the Chinese states (local and national), and the cinchona programme’s paradoxical nature of anti-imperialism and internal colonialism, provides another comparison in East Asia, which will also revise and make dialogues with current dominant narratives in the global history of cinchona. The Hekou cinchona cultivation programme was not originally a colonial/imperial project like those in Western or Japanese empires but was initiated by technocrats in Yunnan in the 1930s to eliminate endemic malaria, as part of local developmental state-building. Moreover, their agenda of achieving quinine self-sufficiency against the Dutch turned out to be anti-imperialist in nature. During the Sino-Japanese War (1937–45), the Hekou programme was integrated into the Chinese central government’s national defence project against the Japanese imperialism, as well as to solve broader wartime epidemic crises in southwest China, together with other state-led, nation-wide bioprospecting programmes for medicinal plants. However, its new reorientation to develop the semi-autonomous malaria-ridden Pusi frontier to provide more war sources indicated that the state-led and anti-imperial cinchona programme had ambiguously served as a tool of internal colonialism.

It also contributes to a recent scholarly interest in global histories of medicine, which highlights the role of networks in the global spread of medical knowledge, personnel and objects.^88^ By illuminating how Yunnan local state-builders were initially rejected by the Dutch, but later on managed to acquire cinchona seeds from British India, French Indochina and the USA via global botanic cinchona cultivation networks, this article draws attention to the importance of collaborations, as well as conflicts in the global history of medical networks.

Last but not the least, it contributes to ongoing revisions of Nationalist regime (1928–49) and particularly adds to the growing body of literature on the pivotal period between 1937 and 1945—when China was at war with the Japanese—a crucial decade that has been understudied until recently.^89^ They have reminded us that at both the central and local levels, the Nationalist regime was not a total failure or ‘an abortive revolution’.^90^ As the history of Hekou programme indicates that there was significant progress made even under difficult wartime circumstances and argues that setbacks in the 1940s were primarily due to war and other factors that could not be anticipated or controlled by both the national and local developmental states.

## Footnotes

1 ‘Dian sheng zhongzhi jinjina shu zhi shiyan’, *Kexue*, 1937, 3, 255–56.

2 Mark Honigsbaum and Merlin Willcox, ‘Cinchona’, in Merlin Willcox, Gerard Bodeker and Philippe Rasoanaivo, eds, *Traditional Medicinal Plants and Malaria* (Boca Raton: CRC Press, 2004), 21–41.

3 Lin Yongxin, ‘Yunnan sheng Hekou shizhi jinji’na zhi jinguo’, *Tai Lin*, 1947, 1, 54–65.

4 Daniel Headrick, *The Tools of Empire: Technology and European Imperialism in the Nineteenth Century* (Oxford: Oxford University Press, 1981).

5 Richard Drayton, *Nature’s Government: Science, Imperial Britain, and the ‘Improvement’ of the World* (New Haven: Yale University Press, 2000).

6 Londa Schiebinger and Claudia Swan, eds, *Colonial Botany: Science, Commerce, and Politics in the Early Modern World* (Philadelphia: University of Pennsylvania Press, 2005).

7 Zhao Xuemin, *Bencao gangmu shiyi* (Supplements to Systematic Materia Medica) (1765. Reprint. Beijing: Renmin weisheng chubanshe, 1963), 208; J. W. Stephens, ‘Pates Medicinales and Quinquina: The Treatment of K’ang Hsi, Emperor of China’, *Journal of Tropical Medicine and Hygiene*, 1937, 40, 187–88; Wang Jiming, ‘Guanyu jinji’na chuanru woguo de jizai’, *Zhonghua yishi zazhi*, 1954, 6, 28–31; Marta Hanson, ‘Jesuits and Medicine in the Kangxi Court (1662–1722)’, *Pacific Rim Report*, 2007, 43, 1–10.

8 For the history of quinine and its global spread, see Andreas-Holger Maehle, *Drugs on Trial: Experimental Pharmacology and Therapeutic Innovation in the Eighteenth Century* (Amsterdam and Atlanta: Editions Rodopi B. V., 1999), 223–309; James L. A. Webb, Jr., *Humanity’s Burden: A Global History of Malaria* (New York: Cambridge University Press, 2009), 92–126. On the introduction and consumption of quinine in China in the nineteeth and twentieth centuries, see Yubin Shen, ‘Malaria and Global Networks of Tropical Medicine in Modern China, 1919–1950’ (unpublished PhD thesis, Georgetown University, 2017), 193–200.

9 Chalmers Johnson, *MITI and the Japanese Miracle: The Growth of Industrial Policy,1925–1975* (Stanford: Stanford University Press, 1982).

10 William Kirby, ‘Engineering China: Birth of the Developmental State, 1928-37’, in Wen-hsin Yeh, ed., *Becoming Chinese: Passages to Modernity and Beyond, 1900–1950* (Berkeley: University of California Press, 2000), 137–60. Also see William C. Kirby, ‘The Nationalist Regime and the Chinese Party-State, 1928–1959’, in Merle Goldman and Andrew Gordon, eds, *Contemporary East Asia in Historical Perspective* (Cambridge: Harvard University Press, 2000), 211–37.

11 For more about Sun Yat-sen and his technocratic and developmentalist ideas, see Mariko Takegami, *Man obu saiensu son bun: Shisoshiteki kosatsu* (Tokyo: Keisoshobo, 2014).

12 Seiichiro Yoshizawa used *the development state* and Jürgen Habermas’s ‘technology and science as ideology’ to discuss the Nationalists’ plan of reconstructing the Northwest region. See Seiichiro Yoshizawa, ‘Nankin kokumin seifu to seihoku kensetsu’, *Gendai Chugoku Kenkyu*, 2006, 29, 18–28; on water control such as Huai River hydraulics, see David A. Pietz, *Engineering the State: The Huai River and Reconstruction in Nationalist China, 1927–1937* (New York: Routledge, 2002). Kenneth Pomeranz recently stated that the developmentalism of the Chinese political economy could be traced back at least as far back as the late imperial period in the 1500s. See Kenneth Pomeranz, ‘The Transformation of the China’s Environment, 1500–2000’, in Edmund Burke III and Kenneth Pomeranz, eds, *The Environment and World History* (Berkeley: University of California Press, 2009), 118–64.

13 One theoretical example is Elizabeth J. Remick, *Building Local States: China during the Republican and Post-Mao Eras* (Cambridge: Harvard University Asia Center, 2004).

14 Emily M. Hill, *Smokeless Sugar: The Death of a Provincial Bureaucrat and the Construction of China’s National Economy* (Vancouver: UBC Press, 2010).

15 Xie Benshu, *Long Yun zhuan* (Chengdu: Sichuan minzu chubanshe, 1988), 94–116. For example, the Yunnan Economic Committee led by the technocrat Miao Jiaming (1894–1988) successfully accomplished a certain level of industrialisation and provided sufficient financial support for Long Yun’s regime by developing and innovating Yunnan’s economic and mining sections with modern technology. See J. C. S. Hall, *The Yunnan Provincial Faction, 1927–1937* (Canberra: Department of Far Eastern History, Australian National University, 1976).

16 On the YAMC (1939–45) as part of the Yunnan local developmental state, see Yubin Shen, ‘Malaria and Global Networks of Tropical Medicine in Modern China, 1919–1950’ (unpublished PhD thesis, Georgetown University, 2017), 123–84.

17 Xiang Guoxiang, Cai Yongshu and Liu Pingshan, ‘Zhang Banhan zhuanlue’, *Zhenxiong wenshi ziliao*, 1983, 1, 102–05.

18 Zhang Banghan, ‘Yunnan sheng jianshe zhuanhao’, *Zhongguo jianshe*, 1934, 8, 85–100.

19 Hill, *Smokeless Sugar*, 21. For a similar discussion on modern agricultural science in China, see Randall Stross, *The Stubborn Earth: American Agriculturalists on Chinese Soil, 1898–1937* (Berkeley: University of California Press, 1986). E. Elena Songster, ‘Cultivating the Nation in Fujian’s Forests: Forest Policies and Afforestation Efforts in China, 1911–1937’, *Environmental History*, 2003, 8, 452–73.

20 After the death of Sun, the university was renamed as Sun Yat-sen University in 1926. Deng Zhiyi, ‘Lun nongxueyuan zhi guoqu yu jianglai’, *Nong shen yuekan*, 1933, 171, 1.

21 Luo Shiming *et al.*, *Hua’nan nongye daxue xiaoshi* (Guangzhou: Guangdong keji chubanshe, 1999).

22 In 1940, Huang published the first monograph on tropical plantations in China. See Huang Riguang, *Zhongguo redai zuowu* (Changshan: Shangwu yinshuguan, 1940).

23 Huang Riguang, ‘Yunnan zhi nong lin jianshe’, *Nong shen yuekan* 1940, 215, 13–20.

24 Zhang Banghan, ‘Yunnan sheng zhi nongye jianshe’, *Zhong nong yuekan*, 1943, 4, 59–64.

25 Yunnan Provincial Archives (hereafter, YPA), 77-23-76, ‘Yunnan jianshe ting zhongzhi jinji’na zhi shikuang, 1940’.

26 Jiangsu sheng Tao Xingzhi yanjiu hui, ed., *Tao Xingzhi rizhi* (Nanjing: Jiangsu jiaoyu chubanshe, 1991), 265.

27 Zheng Zuyou, ‘Yunnan nueji liuxing lishi ji diaoyan qingkuang’, in Zhengxie Yunnan sheng weiyuanhui wenshi ziliao yanjiu weiyuanhui, ed.*, Yunnan wenshi ziliao xuanji 35* (Kunming: Yunnan renmin chubanshe, 1989), 249–71.

28 Huang Riguang, ‘Yunnan zhi nong lin jianshe’, *Nong shen yuekan*, 1940, 215, 13.

29 YPA, 77-23-76, ‘Yunnan Hekou tuiguang zhongzhi jinji’na shu ji shiyan jihua shu, Jan 2, 1940’.

30 Liang Guangshang, *Jinji’na shu zhi zaipei yu yongtu* (Shanghai: Zhengzhong shuju, 1946), 81.

31 Margherita Zanasi, *Saving the Nation: Economic Modernity in Republican China* (Chicago: The University of Chicago Press, 2006).

32 YPA, 77-23-76, ‘Yunnan Hekou tuiguang zhongzhi jinji’na shu ji shiyan jihua shu, January 2, 1940’.

33 Lin Xixun, ‘Yunnan zhi jinji’na’, *Nong Shen*, 1942, 225, 82–83.

34 YPA, 77-23-76, ‘Yunnan jianshe ting zhongzhi jinji’na zhi shikuang, 1940’.

35 For a general introduction on worldwide cinchona cultivation, see Liang Ruxiang, ‘Jinji’na zhi yanjiu’, *Nong bao*, 1947, 12, 34.

36 Kavita Philip, ‘Imperial Science Rescues a Tree: Global Botanic Networks, Local Knowledge and the Transcontinental Transplantation of Cinchona’, *Environment and History*, 1995, 1, 173–200.

37 Lucile H. Brockway, *Science and Colonial Expansion: The Role of the British Royal Botanic Gardens* (New York: Academic Press, 1979), 103–39. Also see Donovan Williams, ‘Clements Robert Markham and the Introduction of the Cinchona Tree into British India, 1861’, *The Geographical Journal*, 1962, 128, 431–442; and Drayton, *Nature’s Government*, 206–11.

38 Andrew Goss, *The Floracrats: State-Sponsored Science and the Failure of the Enlightenment in Indonesia* (Madison: University of Wisconsin Press, 2011), 33–58.

39 Andrew Goss, ‘Building the World’s Supply of Quinine: Dutch Colonialism and the Origins of a Global Pharmaceutical Industry’, *Endeavour*, 2014, 38, 8. On the different historical trajectories of cinchona plantations in the Dutch and British empires, see Arjo Roersch van der Hoogte and Toine Pieters, ‘Science in the Service of Colonial Agro-industrialism: The Case of Cinchona Cultivation in the Dutch and British East Indies, 1852–1900’, *Studies in History and Philosophy of Biological and Biomedical Sciences*, 2014, 47, 12–22.

40 Xixun, ‘Yunnan zhi jinji’na’, 81–82; A. Yersin et A. Lambert, *Essais d’Acclimatation de L’arbre a quinquina en Indochine* (Hanoi: Imprimerie D’Extreme-orient, 1931), 5–11.

41 Jiao Qiyuan, ‘Jinji’na shuang shu ji qita zhi nue zhi yaoyong zhiwu’, *Xi’nan shiye tongxun*, 1942, 5, 18.

42 Nagumo Seiji, ‘Kina no kokunai saibai ni kansuru shiteki kenkyu’, *Yakugaku Zasshi*, 2011, 131, 1527–1543; Ya-wen Ku, ‘Rizhi shiqi Taiwan de jinji’na shu zaipei yu kui’ning zhiyao’, *Taiwan shi yanjiu*, 2011, 18, 47–91.

43 According to Norman Taylor, it was not until 1937 that the Dutch colonial government began to embargo cinchona seeds, and before that many countries successfully purchased cinchona seeds from Java. See Norman Taylor, *Cinchona in Java: The Story of Quinine* (New York: Greenberg, 1945), 77–78. Also see Tanaka Chōzaburō, *Nanpō shokusan shigen ron* (Tokyo: Yokendo, 1943).

44 *Tao Xingzhi rishi*, 265.

45 Lufeng Farm was the oldest tree farm in modern Yunnan, dating back to 1904. See Li Ronggao, ‘Yunnan minguo shiqi linchang kao’, *Nongye kaogu*, 2003, 1, 172. For more about the history of agricultural experiment stations in early twentieth-century China, see Peter Lavelle, ‘Agricultural Improvement at China’s First Agricultural Experiment Stations’, in Denise Philips and Sharon Kingsland, eds, *New Perspectives on the History of Life Sciences and Agriculture* (Springer, 2015), 323–344.

46 For more about Joseph Rock’s botanic adventures in Yunnan, see Erik Mueggler, *The Paper Road: Archive and Experience in the Botanical Exploration of West China and Tibet* (Berkeley: University of California Press, 2011).

47 Xixun, ‘Yunnan zhi jinji’na’, 82–83; Yongxin, ‘Yunnan sheng Hekou shizhi jinji’na zhi jinguo’, 56.

48 ‘Yunnan sheng jianshe zhuanhao’, 101–02.

49 YPA, 77-9-1502, ‘Hekou redai zuowu shiyanchang, 1940’.

50 Yongxin, ‘Yunnan sheng Hekou shizhi jinji’na zhi jinguo’, 56–57.

51 YPA, 77-1-247, ‘Gongwu renyuan zhenbie biao: Zhang Jiliang’.

52 Yongxin, ‘Yunnan sheng hekou shizhi jinji’na zhi jinguo’, 64.

53 YPA, 77-23-93, ‘Jinji’na shu chonghai baogao, 1938’.

54 *Tao Xingzhi rishi*, 265.

55 YPA, 77-23-76, ‘Yunnan jianshe ting zhongzhi jinji’na zhi shikuang, 1940’.

56 Xixun, ‘Yunnan zhi jinji’na’, 83.

57 YPA, 77-11-1967, ‘Hekou zuowu shiyanchang shizhong gezhong jinji zuowu zhongmiao laiwang feiyong kaizhi’.

58 YPA, 77-9-966-02, ‘Yunnan sheng jianshe ting Yi’xi redai zuowu shiyanchang 39 niandu gongzuo baogao’.

59 YPA, 77-32-51, ‘Yi’xi redai zuowu shiyanchang wenti baogao, November 2, 1942’.

60 A similar case of land reclamation and wartime refugees in Shanxi province, see Micah S. Muscolino, ‘Refugees, Land Reclamation, and Militarized Landscapes in Wartime China: Huanglongshan, Shaanxi, 1937–45’, *The Journal of Asian Studies*, 2010, 69, 453–78.

61 Long Yun, ‘Changqi kangzhan yu kaifa Yunnan zhi guanxi xing’, *Yunnan sheng zhengfu gongbao*, 1939, 11, 24–25.

62 Jiang Yingliang, *Sipu yanbian kaifa fang’an* (Kunming: Yunnan sheng minzheng ting bianjiang xingzheng sheji weiyuanhui, 1945), 34.

63 James Scott, *The Art of Not Being Governed: An Anarchist History of Upland Southeast Asia* (New Haven: Yale University Press, 2009).

64 C. Patterson Giersch, *Asian Borderlands: The Transformation of Qing China’s Yunnan Frontier* (Cambridge: Harvard University Press, 2006); David Bello, *Across Forest, Steppe, and Mountain: Environment, Identity, and Empire in Qing China’s Borderlands* (Cambridge: Cambridge University Press, 2016), 169–218.

65 For the still controversial statement of the Tai people’s malaria resistance, see David Bello, ‘To Go Where No Han Could Go for Long: Malaria and the Qing Construction of Ethnic Administrative Space in Frontier Yunnan’, *Modern China*, 2005, 31, 283–317. See also ‘The League of Nations Anti-Epidemic Work in China in 1939’, *Bulletin of the Health Organization*, 1940/41, 3, 256. In 1941, the Yunnan Anti-Malaria Commission conducted an investigation on such issue and found that the local Tai people were indeed more resistant to malaria, compared with the Han Chinese; see Zhou Yimin, ‘Xunhui kangnue dui zai Yuanjiang xian zhi gongzuo gaikuang’, *Yunnan weisheng*, 1942, 12, 10. For a recent study on indigenous resistance to malaria in Africa, see James L. A. Webb, *The Long Struggle Against Malaria in Tropical Africa* (Cambridge: Cambridge University Press, 2014), 45–68.

66 Even today, the southwest frontier/borderland has been still ‘considered’ as a certain site of disease emergence. See Sandra Teresa Hyde, *Eating Spring Rice: The Cultural Politics of AIDS in Southwest China* (Berkeley: University of California Press, 2007).

67 Jiang Yingliang, *Bianjiang xingzheng renyuan shouce* (Kunming: Yunnan sheng minzheng ting bianjiang xingzheng sheji weiyuanhui, 1944), 53.

68 League of Nations Health Organization Malaria Commission, *Principles and Methods of Antimalarial Measures in Europe* (Geneva, 1927), 84.

69 Liang Guangshang, ‘Yunnan jinji’na shu zhi peizhi yu kaifa nuequ’, *Zhongyang ribao* (Kunming edition), 13 April 1940.

70 Huang Riguang, ‘Yunnan shi zhong jinji’na yu kai geng zhang qu zhi guan xi’, *Yishi bao* (Kunming), 16 June (*Kexue fukan*, no.10), 1939.

71 YPA, 77-23-12, ‘Zhang Jiliang zhidao Nanqiao nongchang zhongzhi jinji’na, March 28, 1939’.

72 Scott, *The Art of Not Being Governed*, 3, 12.

73 Bio-prospecting is the process of searching and examining plants to obtain medicinal properties. See Londa Schiebinger, *Plants and Empire: Colonial Bioprospecting in the Atlantic World* (Cambridge: Harvard University Press, 2004); Londa Schiebinger and Claudia Swan, eds, *Colonial Botany: Science, Commerce, and Politics in the Early Modern World* (Philadelphia: University of Pennsylvania Press, 2005); Abena Dove Agyepoma Osseo-Asare, *Bitter Roots: The Search for Healing Plants in Africa* (Chicago: The University of Chicago Press, 2014); Anna Winterbottom, *Hybrid Knowledge in the Early East India Company World* (Palgrave Macmillan, 2016), 150–62.

74 Pratik Chakrabarti, ‘Empire and Alternatives: Swietenia febrifuga and the Cinchona Substitutes’, *Medical History*, 2010, 54, 75–94; Abena Dove Osseo-Asare, *Bitter Roots: The Search for Healing Plants in Africa* (Chicago: The University of Chicago Press, 2014), 131–63.

75 Sean Hsiang-lin Lei, *Neither Donkey nor Horse: Medicine in the Struggle over China’s Modernity* (Chicago: The University of Chicago Press, 2014), 193–221.

76 Liu Fengxiang, ‘Woguo changshan zhongzhi shiyan’, *Nongye tongxun*, 1947, 1, 23–24.

77 YPA, 77-11-1965, ‘Quanguo yishi lianhehui zhi quanguo jingji weiyuanhui han, 1936’.

78 YPA, 77-9-966-02, ‘Yunnan jianshe ting Hekou redai zuowu shiyanchuang 31 niandu gongzuo zong huibao’.

79 YPA, 77-23-76, ‘Yunnan jianshe ting zhongzhi jinji’na zhi shikuang’.

80 YPA, 77-23-76, ‘Yunnan sheng jianshe ting 29 niandu tuiguang zhongzhi jinji’na, sanqi, baiqiangkan jihua’.

81 YPA, 77-9-971, ‘Yunnan sheng zhengfu jianshe ting gongzuo baogao 1941’.

82 YPA, 77-23-104, ‘Zhang Shaoxun kuaiyou dai dian, September 14, 1943’.

83 YPA, 77-23-179, ‘Yunnan sheng jianshe ting Hekou redai zuowu shiyanchang gezhong jingfei yusuan ji gongzuo baokao’.

84 YPA, 77-23-179, ‘Yunnan sheng jianshe ting Hekou redai zuowu shiyanchang gezhong jingfei yusuan ji gongzuo baokao’.

85 Yunnan sheng Hekou Yaozu Zizhixian zhi bianzuan weiyuanhui, *Hekou Yaozu zhizhixian zhi* (Beijing: Sanlian chubanshe, 1994), 213.

86 Recent studies for instance include Matthew James Crawford, *The Andean Wonder Drug: Cinchona Bark and Imperial Science in the Spanish Atlantic, 1630–1800* (Pittsburg: University of Pittsburgh Press, 2016); Arjo Roersch van der Hoogte and Toine Pieters, ‘Quinine, Malaria, and the Cinchona Bureau: Marketing Practices and Knowledge Circulation in a Dutch Transoceanic Cinchona–Quinine Enterprise (1920s–30s)’, *Journal of the History of Medicine and Allied Sciences*, 2016, 71, 197–225; and Rohan Deb Roy, *Malarial Subjects: Empire, Medicine and Nonhumans in British India, 1820–1909* (Cambridge: Cambridge University Press, 2017).

87 Ya-wen Ku, ‘The Development of Cinchona Cultivation and ‘Kina Gaku’ in the Japanese Empire, 1912–45’, in Ts’ui-jung Liu and James John Beattie, eds, *Environment, Modernization and Development in East Asia: Perspectives from Environmental History* (New York, NY: Palgrave Macmillan, 2016), 157–81. Another exception is Timothy Yang, ‘Selling an Imperial Dream: Japanese Pharmaceuticals, National Power, and the Science of Quinine Self-Sufficiency’, *East Asian Science, Technology and Society*, 2012, 6, 101–25.

88 See Mark Harrison, ‘A Global Perspective: Reframing the History of Health, Medicine, and Disease’, *Bulletin of the History of Medicine*, 2015, 89, 639–89.

89 A latest example in the field of social history of medicine is Nicole Elizabeth Barnes, *Intimate Communities: Wartime Healthcare and the Birth of Modern China, 1937–1945* (Oakland, CA: University of California Press, 2018).

90 For the now contested ‘failure narrative’, see Lloyd E. Eastman, *The Abortive Revolution: China under Nationalist Rule, 1927–1937* (Cambridge: Harvard University Press, 1974); Lloyd E. Eastman, *Seeds of Destruction: Nationalist China in War and Revolution, 1937–1949* (Stanford: Stanford University Press, 1984).

## Notes

Yubin Shen is a postdoctoral fellow at the Max Planck Institute for the History of Science, Berlin, Germany. He received his PhD in History from the Department of History, Georgetown University, USA in 2017.

